# Reversible Luminescent Switching Induced by Heat/Water Treatment in a Zero-Dimensional Hybrid Antimony(Ⅲ) Chloride

**DOI:** 10.3390/molecules28041978

**Published:** 2023-02-19

**Authors:** Ying-Chen Peng, Hao-Wei Lin, Sheng-Hua Zhou, Jian-Ce Jin, Ting-Hui Zhuang, Abdusalam Ablez, Ze-Ping Wang, Ke-Zhao Du, Xiao-Ying Huang

**Affiliations:** 1 State Key Laboratory of Structural Chemistry, Fujian Institute of Research on the Structure of Matter, Chinese Academy of Sciences, Fuzhou 350002, China; 2 College of Chemistry, Fuzhou University, Fuzhou 350116, China; 3 Fujian Provincial Key Laboratory of Advanced Materials Oriented Chemical Engineering, Fujian Normal University, Fuzhou 350007, China

**Keywords:** inorganic–organic hybrid material, antimony halide, luminescent switching, photoluminescence, water-sensing

## Abstract

Recently zero-dimensional (0-D) inorganic–organic metal halides (IOMHs) have become a promising class of optoelectronic materials. Herein, we report a new photoluminescent (PL) 0-D antimony(III)-based IOMH single crystal, namely [H_2_BPZ][SbCl_5_]·H_2_O (BPZ = benzylpiperazine). Photophysical characterizations indicate that [H_2_BPZ][SbCl_5_]·H_2_O exhibits singlet/triplet dual-band emission. Density functional theory (DFT) calculations suggest that [H_2_BPZ][SbCl_5_]·H_2_O has the large energy difference between singlet and triplet states, which might induce the dual emission in this compound. Temperature-dependent PL spectra analyses suggest the soft lattice and strong electron–phonon coupling in this compound. Thermogravimetric analysis shows that the water molecules in the lattice of the title crystal could be removed by thermal treatment, giving rise to a dehydrated phase of [H_2_BPZ][SbCl_5_]. Interestingly, such structural transformation is accompanied by a reversible PL emission transition between red light (630 nm, dehydrated phase) and yellow light (595 nm, water-containing phase). When being exposed to an environment with 77% relative humidity, the emission color of the dehydrated phase was able to change from red to yellow within 20 s, and the red emission could be restored after reheating. The red to yellow emission switching could be achieved in acetone with water concentration as low as 0.2 vol%. The reversible PL transition phenomenon makes [H_2_BPZ][SbCl_5_]·H_2_O a potential material for luminescent water-sensing.

## 1. Introduction

Inorganic–organic metal halides (IOMHs) have drawn enormous research attention due to their outstanding optoelectronic properties of high carrier mobility, strong absorption, long carrier diffusion length, and so on [1,2]. These properties are intensively related with their delocalized electrons with large Wannier-type excitations characterized by small binding energies (10–40 meV) [3,4]. Devious choices of organic and inorganic components have made dimensional-reduced IOMHs available, which can exhibit strong structural distortions and significant quantum confinement effects [5,6,7]. Especially, zero-dimensional (0-D) IOMHs show localized structures, bringing faster formation and radiative recombination of excitations, which is beneficial to efficient photoluminescence (PL) [8,9]. In addition, 0-D IOMHs may own soft lattice and strong electron–phonon coupling, thus leading to interesting photoluminescent properties, including broad emission and large Stokes shift [10]. As a result, the applications of photoluminescent 0-D IOMHs have flourished in light-emitting diode [11,12,13,14,15], X-ray scintillator [16,17,18], remote thermography [19,20], optical waveguide [21], anti-counterfeiting [22,23,24,25,26], as well as sensor [27,28,29,30].

Previous works indicate that the PL performances (e.g., emission color, quantum yield) of 0-D IOMHs could be adjusted by supramolecular interactions induced by solvent molecules [17,31,32]. The information can be converted to visible color or optical signals [33], termed as vapochromism, which can be directly discerned by the naked eye [34]. Since the water molecule is always present in environments, such as the atmosphere, the water-molecule-sensing is of vital importance for meteorology, industrial production and human health [35,36,37,38]. Today’s sensors for humidity detection include fiber optic [39], fluorescence [40], quartz crystal microbalance (QCM) [41], and capacitive and resistive ones [42]. Optical-based humidity sensors usually have better sensitivity and can be used in environments with strong electromagnetic interference [43]. Thus far, the water-induced PL vapochromism has been occasionally investigated in 0-D IOMHs. For instance, Tang’s group reports on a 0-D IOMH of PEA_2_MnBr_4_ (PEA = doubly-protonated phenethylamine) that exhibits PL color change from green to pink at 38% relative humidity (RH) [28]; Kuang’s group reports on a 0-D (PPZ)_2_SbCl_7_·5H_2_O (PPZ = doubly-protonated 1-phenylpiperazine) with broad red emission, which could be turned to a yellow-emissive phase by removing water molecules in the crystal lattice and the water-free phase shows a water detecting limit of 1.5 vol% [30]. However, it is still challenging to realize the fast-detecting and lower-detecting limit for water-sensing by IOMHs. Herein, we synthesized a new type of water-containing metal halide single crystal, namely [H_2_BPZ][SbCl_5_]·H_2_O (BPZ = benzylpiperazine). This 0-D antimony(III)-based IOMH exhibits yellow and red emission, respectively, during water insertion and removal. The PL switching can be as fast as 20 s, making the water detection possible.

## 2. Results and Discussion

### 2.1. Crystal Structure

**Figure 1:**
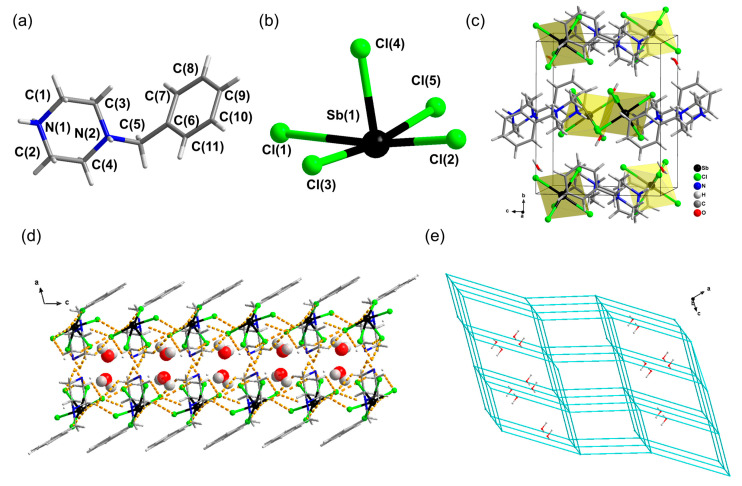
Structural diagrams for the title crystal [H_2_BPZ][SbCl_5_]·H_2_O. A [H_2_BPZ]^2+^ cation (**a**) and a [SbCl_5_]^2−^ anion (**b**) in [H_2_BPZ][SbCl_5_]·H_2_O. (**c**) Unit-cell-packing diagram viewed down the *a*-axis. (**d**) A supramolecular layer in [H_2_BPZ][SbCl_5_]·H_2_O viewed along the *b*-axis in which lattice water molecules are located; water molecules are in CPK mode; hydrogen bonds with water molecules are not shown for clarity. (**e**) Topological net of *pcu* type for anions arrangement in [H_2_BPZ][SbCl_5_]·H_2_O where the lattice water molecules are shown.

The singe-crystal X-ray structure of [H_2_BPZ][SbCl_5_]·H_2_O was determined by SCXRD at 293 K. Crystal data for [H_2_BPZ][SbCl_5_]·H_2_O (C_11_H_20_Cl_5_N_2_OSb, *M* = 495.30 g/mol): monoclinic, space group *P*2_1_/*c* (no. 14), *a* = 15.1814(14) Å, *b* = 12.1122(11) Å, *c* = 10.4217(11) Å, *β* = 103.164(10)°, *V* = 1866.0(3) Å^3^, *Z* = 4, *T* = 293(2) K, *μ*(Mo *K_α_*) = 8.137 mm^−1^, *D*_calc._ = 1.763 g/cm^3^, 4789 reflections measured (5.108° ≤ 2*θ* ≤ 52.254°), 4789 unique (*R*_int_ = 0.0641, *R*_sigma_ = 0.0941) were used in all calculations (Table S1). The final *R*_1_ was 0.0411 (*I* > 2*σ*(*I*)), and w*R*_2_ was 0.0777 (all data). There is one formular unit in the asymmetric unit (Figure S1); that is, it consists of one [H_2_BPZ]^2+^ cation (Figure 1a), one [SbCl_5_]^2−^ anion (Figure 1b), and one water molecule. The inorganic [SbCl_5_]^2−^ anions are separated and charge-balanced by organic [H_2_BPZ]^2+^ cations, forming a 0-D hybrid crystal structure (Figure 1c). In the pyramid-like inorganic anionic unit, the lengths of Sb−Cl bonds range from 2.3823(11) to 2.8865(13) Å, and the angles of Cl−Sb−Cl range from 84.20(4)° to 91.60(5)° (Table 1), which are comparable to those in the literature [44]. Notably, there are abundant H-bonds (N−H···Cl and C−H···Cl H-bonds) among cations and anions, as listed in Table 2, which form a supramolecular layer structure, as shown in Figure 1d and Figure S2, where the lattice water molecules are located and form additional hydrogen bonds with Cl^−^ ions of the anions within the layer (Table 2). As shown in Figure 1e, the anions-packing adopts a topology of *pcu* (primitive cubic net) with a little distortion because of the low symmetry of structure and the distortion of the [SbCl_5_]^2−^ polyhedrons. Noteworthy is that the water molecules are located around the center of cubes built by eight [SbCl_5_]^2−^ units in the topology framework (Figure 1e).

In previous reports, it was found that ionic metal halide crystals could be formed by combining penta-coordinated antimony halide units of [SbCl_5_]^2−^ with monoprotonated 4-benzylpiperidine cations (bpzpipn), a cation with a similar structural geometry but different charge with doubly protonated benzylpiperazine of [H_2_BPZ]^2−^ in [H_2_BPZ][SbCl_5_]·H_2_O. The obtained compounds ([bzpipn]_2_SbCl_5_ [45] and [bzpipn]_2_SbCl_5_·H_2_O [46]) both feature a pseudo 1D chain-like structure of [SbCl_5_]*_n_* due to the presence of an additional secondary Sb···Cl bond in between adjacent [SbCl_5_] units with bond lengths ranging from 3.2182 to 3.3028 Å. By contrast, in the title [H_2_BPZ][SbCl_5_]·H_2_O, all the [SbCl_5_] units are discrete with a shortest Sb···Sb distance of 7.605 Å. The difference in the arrangements of [SbCl_5_] units in these compounds is probably related to the ability of cations of forming N−H···Cl H-bonds; note that [H_2_BPZ] contains a NH_2_ and a NH in the piperazine ring, while [bzpipn] contains a NH_2_ group only in the piperidine ring. Thus, the structural comparison made here highlights the importance of organic cations in constructing ionic metal halides with desired structure and property.

### 2.2. Photophysical Properties

**Figure 2:**
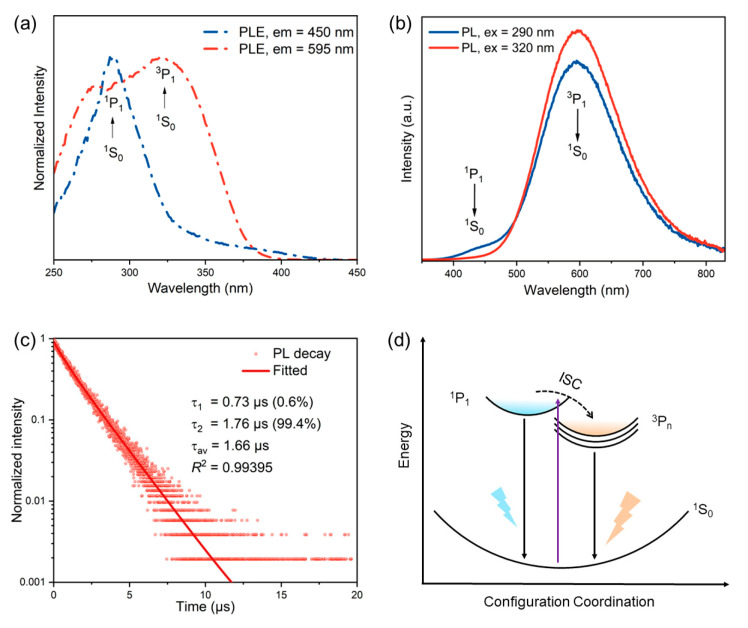
Steady-state and time-resolved PL spectra of [H_2_BPZ][SbCl_5_]·H_2_O at RT. (**a**) The steady-state PLE spectra of [H_2_BPZ][SbCl_5_]·H_2_O, measured with emission wavelengths at 450 and 595 nm, respectively. (**b**) The steady-state PL spectra of [H_2_BPZ][SbCl_5_]·H_2_O with excitation wavelengths at 290 and 320 nm, respectively. (**c**) Time-resolved PL spectrum of [H_2_BPZ][SbCl_5_]·H_2_O at 595 nm emission. The PL lifetime is fitted, calculated, and labelled. (**d**) The proposed PL mechanism in the configuration coordinate diagram.

To characterize the optical properties of [H_2_BPZ][SbCl_5_]·H_2_O, the steady-state and time-resolved PL spectra were measured. The steady-state PL excitation spectrum (emission = 595 nm) shows two peaks at about 265 nm and 320 nm, suggesting ^1^S_0_ → ^1^P_1_ and ^1^S_0_ → ^3^P_1_ electronic transitions in Sb^3+^ ions, respectively (Figure 2a) [47]. The ^1^S_0_ → ^1^P_1_ electronic transition is allowed, while the ^1^S_0_ → ^3^P_1_ is partially allowed by the spin–orbit coupling [48,49]. However, the steady-state PL excitation spectrum (emission = 450 nm) only shows one peak at around 275 nm, suggesting that the ^1^S_0_ → ^3^P_1_ electronic transition nearly disappeared. Under the excitation of 290 nm, [H_2_BPZ][SbCl_5_]·H_2_O exhibits dual-band broad emission peaking at 450 and 595 nm. The dual-band emission could be attributed to the exciton relaxation of ^1^P_1_ → ^1^S_0_ and ^3^P*_n_* → ^1^S_0_, respectively (Figure 2b). However, there is only one broad mono-band yellow emission under the excitation of 320 nm (Figure 2b); this triplet emission shows a peak centered at 595 nm with a Stokes shift of 275 nm. The title compound delivers a PLQY value of 14.33%, which is moderate among this class of compounds [26,30,31,49]. The time-resolved PL spectrum is shown in Figure 2c and utilized to calculate the PL lifetime of the title compound (Figure 2c). The lifetime of [H_2_BPZ][SbCl_5_]·H_2_O can be fitted well via the biexponential function (Equation (1)) [50]:

(1) $$\begin{array}{c} I=A_{1}\exp\left(-t/\tau_{1}\right)+A_{2}\exp\left(-t/\tau_{2}\right) \end{array}$$

The average lifetime can be calculated and obtained by the following Equation (2) [51]:

(2) $$\begin{array}{c} \tau_{av}=\left(A_{1}\tau_{1}{}^{2}+A_{2}\tau_{2}{}^{2}\right)/\left(A_{1}\tau_{1}+A_{2}\tau_{12}\right) \end{array}$$

The lifetime of [H_2_BPZ][SbCl_5_]·H_2_O can be fitted as 1.66 μs, which confirms the triplet emission.

After analyzing the steady-state and time-resolved PL spectra of the title compound, the PL mechanism is proposed as shown in Figure 2d [40]. Under excitation, the electrons in ground state ^1^S_0_ orbitals are excited to the singlet excited state ^1^P_1_ and triplet excited state ^3^P*_n_* orbitals. The intersystem crossing (ISC) from the singlet to triplet orbitals results in the strong triplet emission during the electronic relaxation.

**Figure 3:**
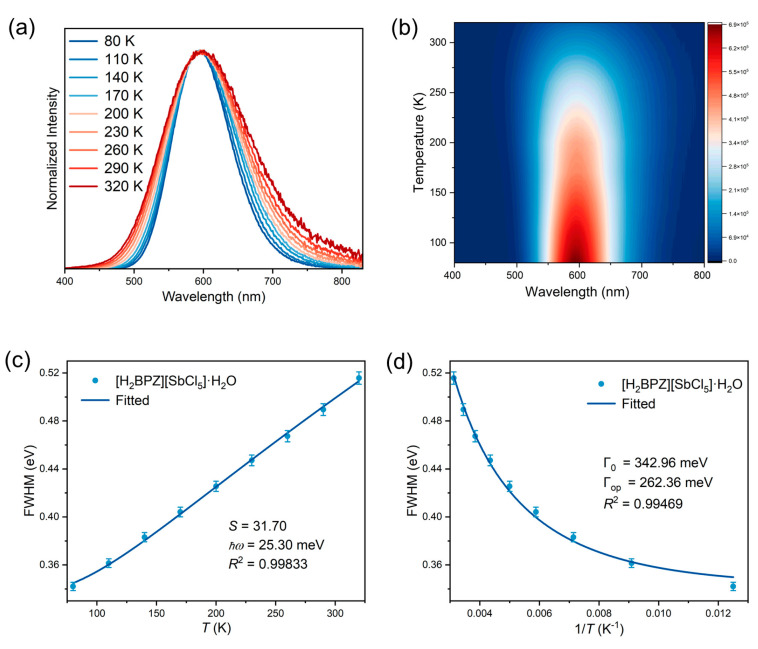
Temperature-dependent PL spectra and fitted physical parameters. Temperature-dependent PL spectra (**a**) and contour map (**b**) of [H_2_BPZ][SbCl_5_]·H_2_O under the excitation of 320 nm. (**c**) Full width at half maximum (FWHM) vs. temperature (*T*) fitted by Equation (3). (**d**) FWHM vs. 1/*T* fitted by Equation (4).

Furthermore, to explore the origin of the broad emission and large Stokes shift of the title compound, temperature-dependent PL spectra ranging from 80 K to 320 K were collected under an excitation of 320 nm. As shown in Figure 3a, b, the title compound shows weaker PL intensity and broadening of the emission band along with the increasing temperature. These temperature-dependent performances are comprehensible. Typically, with increasing temperature, there is enhancement of thermal vibrations resulting in a thermal quenching of PL. Whereafter, the temperature-dependent PL spectra under the 320 nm excitation are further analyzed to obtain several important physical parameters, including Huang–Rhys factor (*S*) and electron–phonon coupling energy (Γ_op_). The *S* can be obtained by fitting the curve of FWHM vs. *T* using the following formula (Equation (3)):

(3) $$\begin{array}{c} \mathrm{FWHM}=2.36\sqrt{S}\hbar \omega \sqrt{\coth\left(\frac{\hbar \omega }{2kT}\right)} \end{array}$$

where FWHM represents full width at half maximum, *ℏ* is Planck constant, *ω* is the phonon frequency, *k* is the Boltzmann constant, and *T* is temperature [52]. The *S* is fitted as 31.70 for [H_2_BPZ][SbCl_5_]·H_2_O (Figure 3c), which is much higher than that of CsPbBr_3_ (*S* = 3.22) [53] and a little higher than that of [PPh_3_H]_2_[SbCl_5_] (*S* = 26.91; PPh_3_ = triphenylphosphine) [49], *S* represents the hardness or softness of the crystal lattice. A small *S* value represents a hard crystal lattice, which is unfavorable for electron–phonon coupling under excitation [53].

To further discuss electron–phonon coupling interactions, the Toyokawa equation (Equation (4)) is used to fit the temperature-dependent PL FWHM:

(4) $$\begin{array}{c} \Gamma \left(T\right)=\Gamma_{0}+\frac{\Gamma_{\mathrm{op}}}{e^{\hbar \omega /kT}-1} \end{array}$$

where Γ_0_ represents the intrinsic line width at absolute 0 K (replaced by the data at 80 K in this work), and Γ_op_ is the electron–phonon coupling energy [54]. The Γ_op_ is fitted as 262.36 meV, larger than that of [DMPZ]_2_SbCl_6_∙Cl∙(H_2_O)_2_ (DMPZ = doubly protonated *N*, *N*′-dimethylpiperazine; Γ_op_ = 65.65 meV) [14] and [PPh_3_H]_2_[SbCl_5_] (Γ_op_ = 144.72 meV) [49], indicating strong electron–phonon coupling in the title compound under the excitation (Figure 3d). Overall, the temperature-dependent PL spectra analysis suggests the soft lattice and strong electron–phonon coupling in the title compound.

### 2.3. Theortical Calculations

**Figure 4:**
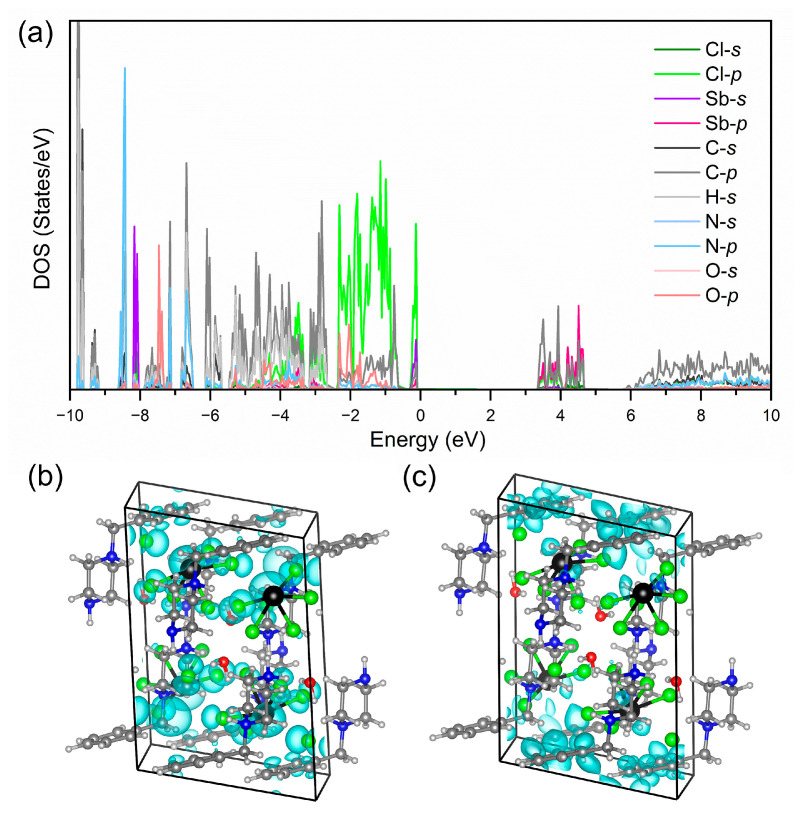
(**a**) The calculated orbital-resolved DOSs of [H_2_BPZ][SbCl_5_]·H_2_O. The highest occupied molecular orbital (HOMO; (**b**)) and lowest occupied molecular orbital (LUMO; (**c**)) along the *ac* plane of [H_2_BPZ][SbCl_5_]·H_2_O.

Density functional theory (DFT) calculations were performed to investigate the band structure and photoluminescent mechanism of [H_2_BPZ][SbCl_5_]·H_2_O. As shown in Figure S5, the title compound shows a calculated direct band gap of 3.45 eV, which is very close to the experimental one of 3.25 eV (Figures S3 and S4). The DOS shows that the valence-band maximum (VBM) is mainly contributed by Sb 5*s* and Cl 3*p* and the conduction-band minimum (CBM) is mostly contributed by Sb 5*p*, C 3*s* and 3*p* (Figure 4a). The nearly dispersionless VBM indicates negligible electronic coupling between inorganic [SbCl_5_]^2−^ units; that is, the title compound behaves a localized electronic structure [55]. Accordingly, the highest occupied molecular orbital (HOMO) is occupied by the inorganic moiety of [SbCl_5_]^2−^ mostly. The electronic cloud was round-like referring to the *s* electrons for Sb atom and spindle-like referring to the p electrons for Cl atom (Figure 4b). The lowest occupied molecular orbital (LUMO) is occupied by Sb atoms and conjugate electrons in benzene rings in organic [H_2_BPZ]^2+^ cations (Figure 4c). The spindle-like electronic cloud of p electrons of Sb atoms is clear. These results suggest the large energy difference between singlet and triplet states, which might induce the dual emission in the title compound [56].

### 2.4. Powder X-ray Diffraction and Thermogravimetric Analysis

**Figure 5:**
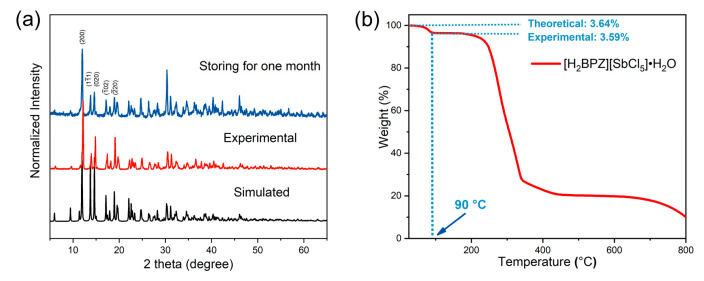
(**a**) The experimental PXRD patterns for as-made [H_2_BPZ][SbCl_5_]·H_2_O and that storing at ambient conditions for one month compared with the one simulated from SCXRD data. (**b**) The TG curve for [H_2_BPZ][SbCl_5_]·H_2_O; the theoretical and experimental weight losses of water molecules in the crystal lattice were calculated and labelled.

The purity and the stability of the title compound were measured by powder X-ray diffraction (PXRD) and thermogravimetric (TG) analysis, as shown in Figure 5. The experimental PXRD pattern of the [H_2_BPZ][SbCl_5_]·H_2_O powders obtained by grinding the crystals is in agreement with the simulated one (Figure 5a), suggesting the purity and uniformity of the as-synthesized sample. Of note is that the as-synthesized crystals of [H_2_BPZ][SbCl_5_]·H_2_O could be steadily stored under ambient conditions for a long time (e.g., one month), as verified by PXRD (Figure 5a). The result implies that the water molecular is stable in the crystal lattice and no phase-transition would happen at ambient conditions. The moderate steric hindrance of organic cations can construct a 2D supramolecular framework, which endows [H_2_BPZ][SbCl_5_]·H_2_O with superior stability. TG analysis indicates a two-step decomposition from RT to 800 °C for [H_2_BPZ][SbCl_5_]·H_2_O. The first weight loss is shown in about 60 to 90 °C (experimental: 3.59% vs. theoretical: 3.64%), implying the removal of one water molecule per formula for [H_2_BPZ][SbCl_5_]·H_2_O (Figure 5b). That means the chemical formula is [H_2_BPZ][SbCl_5_] for the dehydrated phase. However, the corresponding PXRD pattern after losing water molecules differs considerably from the one of the pristine and the simulated one (Figure S6), implying a slightly changed ionic structure after the loss of water molecules [24]. The second weight loss from 90 to 340 °C corresponds to a total decomposition of the title compound.

### 2.5. Luminescent Water-Sensing

**Figure 6:**
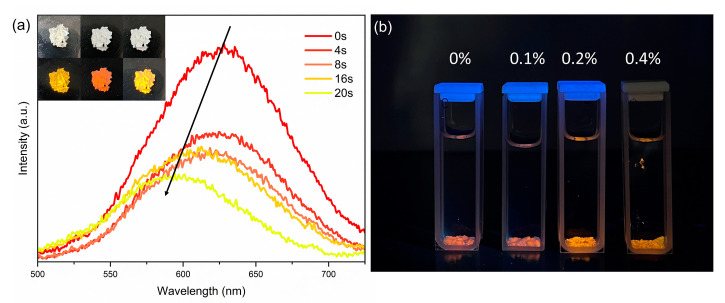
(**a**) The in situ PL spectra for humidity-sensing application; the red-emissive [H_2_BPZ][SbCl_5_] can be transformed to yellow-emissive [H_2_BPZ][SbCl_5_]·H_2_O within 20 s under the ambient condition. Inset: the photographs of [H_2_BPZ][SbCl_5_]·H_2_O power under ambient light (top) and UV light (bottom); from left to right: freshly prepared crystals, samples after heating at 100 °C for 30 min, dehydrated samples placed in ambient condition. (**b**) Dehydrated [H_2_BPZ][SbCl_5_] in acetone solvent containing different amounts of water (0–0.4% *v*/*v*).

The water molecules could be removed by heat treatment of the title compound according to the TG analysis (Figure 5b). Thus, we have performed the dehydration of the title compound by heating at 100 °C for 30 min. After dehydration, the single crystals show pulverization and are not transparent anymore. Moreover, the PL emission has been changed from yellow to red. Interestingly, the PL of the dehydrated sample could be recovered in ambient conditions (average 21 °C and 77% humidity in Fuzhou, China) quickly (inset of Figure 6a). To further characterize the PL emission switching, in situ PL spectra were performed for the dehydrated [H_2_BPZ][SbCl_5_] at ambient conditions. As shown in Figure 6a, the dehydrated [H_2_BPZ][SbCl_5_] exhibits an emission peak at around 630 nm, while the emission peak shows a blue shift becoming 595 nm after 20 s. In addition, the dehydrated [H_2_BPZ][SbCl_5_] exhibits stronger PL intensity than [H_2_BPZ][SbCl_5_]·H_2_O. Quick and distinct PL emission switching make luminescent water-sensing application possible. Then, we have utilized the dehydrated [H_2_BPZ][SbCl_5_] to trace the water content in an organic solvent. Here, the dehydrated powder was soaked into acetone with different water contents (From 0–0.4% *v*/*v*). As the water concentration increased from 0.1 vol% to 0.2 vol%, the red-emissive compound turned to emit yellow light. The results show a detection limit of ca. 0.2 vol% for luminescent humidity-sensing for [H_2_BPZ][SbCl_5_], which is lower than that of another 0-D Sb^3+^-based IOMH of (PPZ)_2_SbCl_7_·5H_2_O (1.5 vol%) [30].

## 3. Materials and Methods

**Reagents:** Antimony(III) oxide (Sb_2_O_3_, RG) was purchased from Adamas Reagent Co. Ltd. (Shanghai, China) Benzylpiperazine (BPZ, 99%) was purchased from J&K Scientific Reagent Co., Ltd. (Beijing, China) Hydrochloric acid solution (HCl, 37%) was purchased from Sinopharm Chemical Reagent Co., Ltd. (Shanghai, China). All reagents and solvents were used without further purification.

**Synthesis of [H_2_BPZ][SbCl_5_]·H_2_O:** 1.0 mmol Sb_2_O_3_ (0.2195 g), 2 mmol BPZ (0.3452 g) and 3 mL HCl (37%) were mixed in a 28 mL Teflon-lined steel autoclave. Then, the reactor was heated at 120 °C for 3 days and naturally cooled to ambient temperature in 5 h. The colorless transparent liquid was obtained and then transported into a 20 mL glass bottle. Finally, transparent colorless prismatic crystals of [H_2_BPZ][SbCl_5_]·H_2_O were crystallized overnight. The product yield was 0.3616 g (73% based on Sb).

**Single-Crystal X-Ray Diffraction (SCXRD):** A suitable single crystal was selected under an optical microscope for SCXRD measurement. Intensity data were collected on a Supernova CCD diffractometer using graphite-monochromated Mo *K*_α_ radiation (λ = 0.71073 Å) at 293 K. The structure was solved by direct methods and refined by full-matrix least-squares on *F*^2^ using the SHELX-2018 program package [57]. The non-hydrogen atoms were refined anisotropically. The hydrogen atoms in the [H_2_BPZ]^2+^ cations were located at geometrically calculated positions, while those of the lattice water molecule were located from difference-Fourier maps and their atomic positions were refined. The crystallographic data and details for structural refinements are listed in Table S1. Selected bond lengths and angles are listed in Table 1. The hydrogen-bonding data are listed in Table 2. CCDC No. 2220612 contains the supplementary crystallographic data for this paper. The data can be obtained free of charge from The Cambridge Crystallographic Data Centre via www.ccdc.cam.ac.uk/data_request/cif (accessed on 18 November 2022).

**Fourier Infrared Spectroscopy (FTIR):** FTIR spectrum was measured by an instrument of Vertex 70 FTIR. Detailed data are shown in Figure S7.

**Powder X-Ray Diffraction (PXRD):** The experimental PXRD patterns were measured by a Rigaku Miniflex-II diffractometer by utilizing Cu *K_α_* radiation (*λ* = 1.54178 Å) at 30 KV and 15 mA in the angular range of 2*θ* = 5–65°. The experimental PXRD pattern after the loss of water molecules were measured by the X-ray diffractometer with D8 Advance made by Bruker at 40 KV and 40 mA in the angular range of 2*θ* = 5–65°. The simulated PXRD pattern was calculated using the SCXRD data via Mercury software.

**Thermogravimetric Analysis (TGA):** TG curve was recorded on a NETZSCH STA 449F3 instrument with a heating rate of 10 K min^−1^ under a dry N_2_ atmosphere.

**Solid-State UV-Visible Absorption Spectroscopy (UV-vis):** The solid-state diffuse reflectance data were recorded on a Shimadzu 2600 UV-vis spectrometer at room temperature (RT) in the range of 800–200 nm. The BaSO_4_ plate was utilized as a standard that possesses 100% reflectance. The absorption data were then obtained from the reflectance spectrum using the Kubelka–Munk function *α*/*S* = (1 − *R*)^2^/2*R*, where *α* refers to the absorption coefficient, *S* refers to the scattering coefficient and *R* refers to the reflectance [58]. The test was performed on the solid-state sample in polycrystalline form.

**Steady-State Photoluminescence Spectra:** The photoluminescence excitation (PLE), photoluminescence (PL) spectra and photoluminescent quantum yield (PLQY) were measured on the FLS1000 UV/V/NIR fluorescence spectrometer. The excitation light source is a solid picosecond diode exciter with a pulse width of 57 picoseconds. The tests were performed on solid-state samples in polycrystalline form.

**Time-Resolved Photoluminescence Spectra:** Time-resolved PL spectra were measured on the FLS1000 UV/V/NIR fluorescence spectrometer. The tests were performed on solid-state samples in polycrystalline form.

**Temperature-Dependent Photoluminescence Spectra:** Temperature-dependent PL spectra were measured on the FLS980 fluorescence spectrometer ranging from 80 K to 320 K. The tests were performed on the samples in polycrystalline form.

Density functional theory (DFT) calculations: DFT calculations were implemented in the Vienna Ab initio Simulation Package (VASP) [59,60,61]. A generalized gradient approximation (GGA) for the exchange-correlation term with Perdew–Burke–Ernzerhof (PBE) exchange–correlation functional was applied for the electron–electron exchange–correlation processes. The projected augmented wave (PAW) potentials were used with the valence states 2*s*, 2*p* for C and N; 3*s*, 3*p* for Cl and 5*s*, 5*p* for Sb, respectively. To ensure sufficient accuracy, the Brillouin zone was implemented by a Monkhorst–Pack k-point mesh of 3 × 3 × 5, and a high cut-off energy of 500 eV for the plane wave expansion was chosen. The self-consistent field (SCF) computations were set to a convergence criterion of 1 × 10^−5^ eV and the force criterion was 0.02 eV/Å. The Fermi level (EF = 0 eV) was chosen as the reference of the energy.

## 4. Conclusions

In summary, a new PL 0-D IOMH of [H_2_BPZ][SbCl_5_]·H_2_O has been studied in this work. The structure of this water-containing metal halide single crystal was determined in detail. The photophysical dynamics were investigated by temperature-dependent PL spectra and DFT calculations. The yellow emission of [H_2_BPZ][SbCl_5_]·H_2_O originates from the recombination of singlet/triplet dual-band emission. DFT calculations show that the VBM and CBM are mostly located at the [SbCl_5_]^2−^ unit, resulting in a large energy difference between the singlet and triplet states. Several important parameters, including *S* Γ_op_, have been fitted from temperature-dependent PL spectra. The results reveal that the large FWHM of PL should be owing to the soft lattice and strong electron–phonon coupling. It is worth noting that the water molecules in the [H_2_BPZ][SbCl_5_]·H_2_O structure can be removed by heating, which causes the luminescent color change from yellow to red. The removed water molecules can be quickly restored to the material in the high RH environment or solution with water concentration as low as 0.2 vol%. This study not only provides a new type of lead-free metal halide-based emitter, but also paves the way for designing new PL metal halide materials for humidity-sensing.

## Figures and Tables

**Table 1:** Select bond lengths (Å) and bond angles (°) for [H_2_BPZ][SbCl_5_]·H_2_O at 293 K.

Sb(1)-Cl(4)	2.3823(11)	Sb(1)-Cl(5)	2.6675(13)
Sb(1)-Cl(2)	2.4585(12)	Sb(1)-Cl(1)	2.8865(13)
Sb(1)-Cl(3)	2.5478(14)		
Cl(4)-Sb(1)-Cl(2)	88.39(4)	Cl(2)-Sb(1)-Cl(5)	90.78(5)
Cl(4)-Sb(1)-Cl(3)	88.05(4)	Cl(4)-Sb(1)-Cl(1)	83.65(4)
Cl(2)-Sb(1)-Cl(3)	91.60(5)	Cl(3)-Sb(1)-Cl(1)	88.93(5)
Cl(4)-Sb(1)-Cl(5)	84.20(4)	Cl(5)-Sb(1)-Cl(1)	87.63(4)

Symmetry transformations used to generate equivalent atoms: *n/a*.

**Table 2:** Hydrogen bonds for [H_2_BPZ][SbCl_5_]·H_2_O at 293 K.

D-H···A	*d*(D-H)	*d*(H···A)	*d*(D···A)	<(DHA)
N(1)-H(1B)···Cl(1)	0.89	2.38	3.184(4)	151.0
N(1)-H(1B)···Cl(4)	0.89	2.67	3.210(4)	120.1
N(1)-H(1A)···O(1)	0.89	1.98	2.843(5)	163.8
N(2)-H(2)···Cl(5)#1	0.98	2.14	3.106(4)	166.9
C(1)-H(1C)···Cl(5)#1	0.97	2.90	3.632(5)	133.0
C(2)-H(2A)···Cl(5)#2	0.97	2.88	3.463(5)	119.6
C(2)-H(2B)···Cl(4)#2	0.97	2.98	3.748(4)	136.6
C(4)-H(4B)···Cl(3)#3	0.97	2.79	3.703(5)	158.0
C(4)-H(4B)···Cl(4)#3	0.97	2.91	3.594(5)	128.0
C(4)-H(4A)···Cl(2)#2	0.97	2.83	3.690(5)	148.3
C(4)-H(4A)···Cl(5)#2	0.97	2.81	3.463(4)	125.2
O(1)-H(1E)···Cl(3)#3	0.818(10)	2.67(3)	3.408(4)	152(6)
O(1)-H(1F)···Cl(1)#4	0.820(10)	2.393(12)	3.210(4)	175(6)

Symmetry transformations used to generate equivalent atoms: #1 −*x* + 1, −*y* + 1, −*z* #2 −*x* + 1, *y* − 1/2, −*z* + 1/2 #3 −*x* + 1, −*y* + 1, −*z* + 1 #4 −*x* + 1, *y* + 1/2, −*z* + 1/2.

## Data Availability

All the available data are incorporated in the MS.

## Supplementary Materials

The following supporting information can be downloaded at: https://www.mdpi.com/article/10.3390/molecules28041978/s1. Table S1: Crystal data and structure refinement details for [H_2_BPZ][SbCl_5_]·H_2_O at 293 K; Figure S1: *ORTEP* drawing (50% ellipsoid probability) of the asymmetric unit of [H_2_BPZ][SbCl_5_]·H_2_O at 293 K; Figure S2: A supramolecular layer in [H_2_BPZ][SbCl_5_]·H_2_O viewed along the *a*-axis in which lattice water molecules are located; water molecules are in CPK mode; hydrogen bonds with water molecules are not shown; Figure S3: Solid-state UV-visible absorption spectrum of [H_2_BPZ][SbCl_5_]·H_2_O at RT; Figure S4: The experimental direct band gap of [H_2_BPZ][SbCl_5_]·H_2_O calculated as 3.25 eV; Figure S5: The band structure of [H_2_BPZ][SbCl_5_]·H_2_O and the direct band gap is calculated as 3.45 eV; Figure S6: The simulated (bottom) and experimental (middle) PXRD patterns for [H_2_BPZ][SbCl_5_]·H_2_O, and experimental PXRD pattern for [H_2_BPZ][SbCl_5_] (top); Figure S7: FTIR spectrum of [H_2_BPZ][SbCl_5_]·H_2_O crystal powder at RT.
