# Supplementary material for: Reversible Luminescent Switching Induced by Heat/Water Treatment in a Zero-Dimensional Hybrid Antimony(Ⅲ) Chloride
Source: Molecules. 2023 Feb 19;28(4):1978. doi: 10.3390/molecules28041978 (PMC9965921; doi:10.3390/molecules28041978)
Supplement: Supplementary file 1 [file molecules-28-01978-s001.zip › molecules-2216089-supplementary.pdf]

Electronic Supporting Information:

Reversible Luminescent Switching Induced by Heat/Water Treatment in a Zero-Dimensional Hybrid Antimony(III) Chloride

Ying-Chen Peng [1,†], Hao-Wei Lin [1,2,†], Sheng-Hua Zhou [1,†], Jian-Ce Jin [1], Ting-Hui Zhuang [1,3], Abdusalam Ablez [1,2], Ze-Ping Wang [1], Ke-Zhao Du [3] and Xiao-Ying Huang [1,*]

[1] State Key Laboratory of Structural Chemistry, Fujian Institute of Research on the Structure of Matter, Chinese Academy of Sciences, Fuzhou 350002, China
[2] College of Chemistry, Fuzhou University, Fuzhou 350116, China
[3] Fujian Provincial Key Laboratory of Advanced Materials Oriented Chemical Engineering, Fujian Normal University, Fuzhou 350007, China
* Correspondence: xyhuang@fjirsm.ac.cn, Fax: +86-591-6317-3145
† These authors contributed equally to this work.

Table S1. Crystal data and structure refinement details for $[H_2BPZ][SbCl_5]\cdot H_2O$ at 293 K.

CCDC number	2220612
Empirical formula	$C_{11}H_{20}Cl_5N_2OSb$
Formula weight	495.29
Temperature/K	293(2) K
Wavelength/Å	0.71073
Crystal system	monoclinic
Space group	$P2_1/c$
a/Å	15.1814(14)
b/Å	12.1122(11)
c/Å	10.4217(11)
α/°	90
β/°	103.164(10)
γ/°	90
V/Å^3	1866.0(3)
Z	4
ρ_{calc} g/cm^3	1.763
Absorption coefficient/mm^{-1}	2.191
F(000)	976
Crystal size/mm^3	0.200 × 0.100 × 0.050
Theta range for data collection /°	2.174 – 29.729
Limiting indices	$-20 \leq h \leq 20$, $-16 \leq k \leq 16$, $-12 \leq l \leq 12$
Reflections collected/ unique	4789/4789
Completeness to theta	25.242, 100.0 %
Absorption correction	Semi-empirical from equivalents
Refinement method	Full-matrix least-squares on F^2
Data/restraints/parameters	4789/2/189
Goodness-of-fit on F^2	1.074
Final R indexes [$I >= 2\sigma(I)$]	$R^1 = 0.0411$, $wR^2 = 0.0722$
Final R indexes [all data]	$R^1 = 0.0785$, $wR^2 = 0.0889$
Extinction coefficient	0.00159(18)
Largest diff. peak and hole/e Å^{-3}	0.752, -0.778

[a] $R_1 = \sum \| F_o | - | F_c \| / \sum | F_o |$, [b] $wR_2 = [\sum w(F_o^2 - F_c^2)^2 / \sum w(F_o^2)^2]^{1/2}$

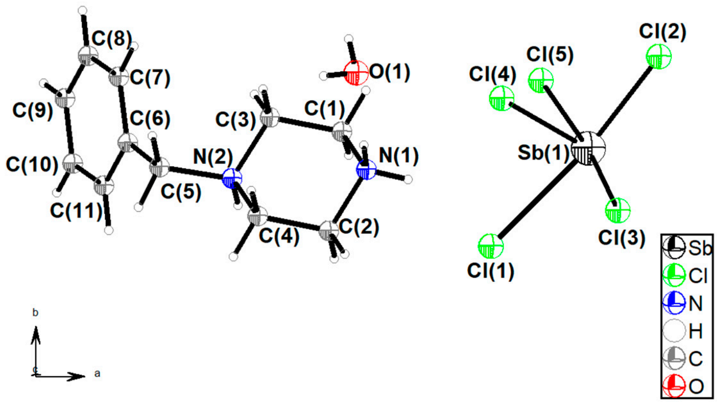

Figure S1. *ORTEP* drawing (50% ellipsoid probability) of the asymmetric unit of $[H_2BPZ][SbCl_5]\cdot H_2O$ at 293 K.

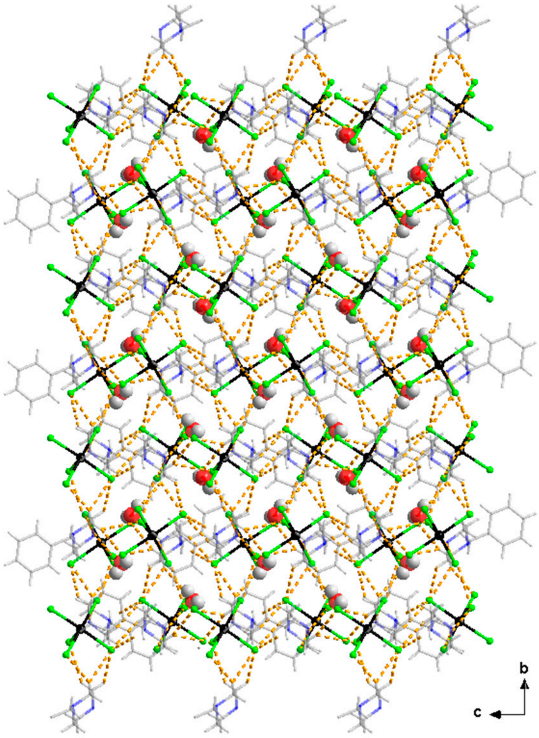

Figure S2. A supramolecular layer in $[H_2BPZ][SbCl_5]\cdot H_2O$ viewed along the *a* axis in which lattice water molecules are located; water molecules are in CPK mode; hydrogen bonds with water molecules are not shown.

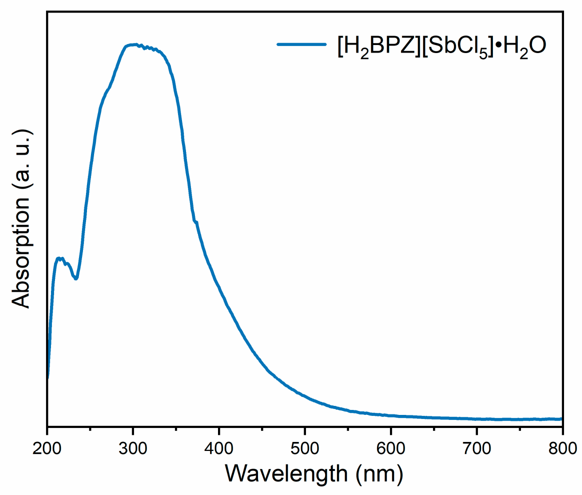

Figure S3. Solid-state UV-visible absorption spectrum of $[H_2BPZ][SbCl_5]{\bullet}H_2O$ at RT.

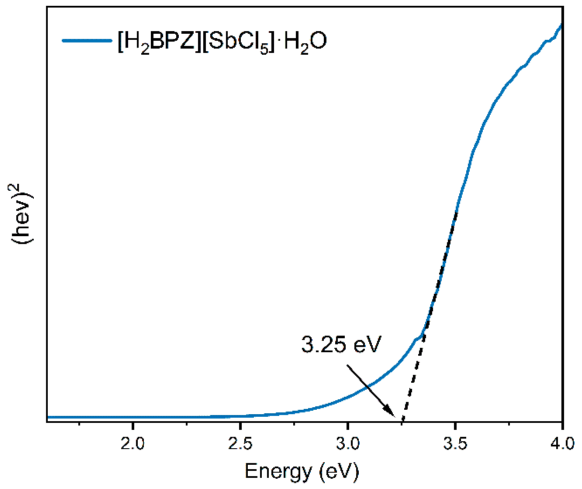

Figure S4. The experimental direct band gap of $[H_2BPZ][SbCl_5]{\bullet}H_2O$ calculated as 3.25 eV.

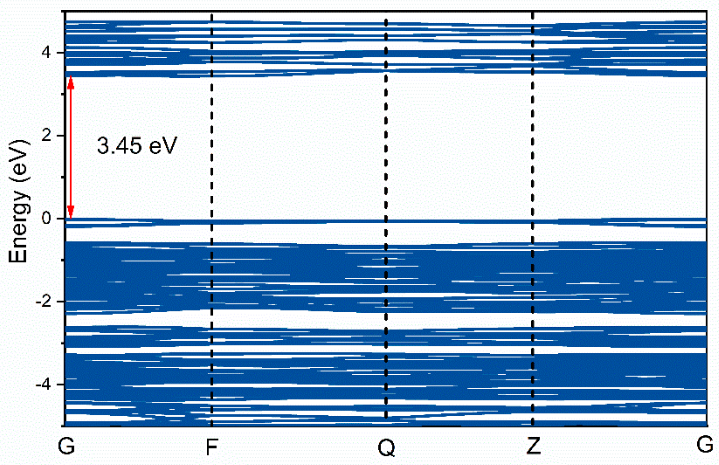

Figure S5. The band structure of $[H_2BPZ][SbCl_5]•H_2O$ and the direct band gap is calculated as 3.45 eV.

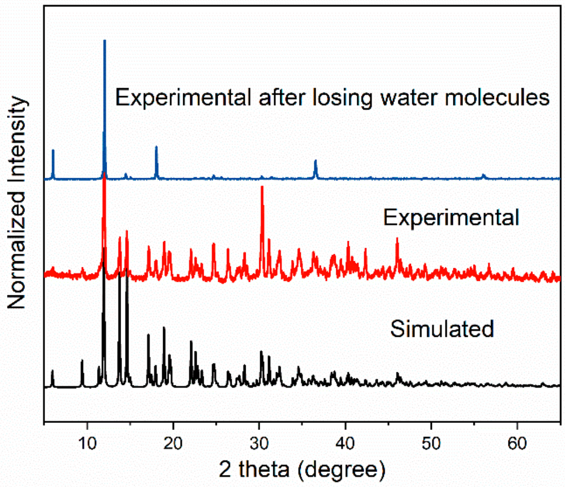

Figure S6. The simulated (bottom) and experimental (middle) PXRD patterns for $[H_2BPZ][SbCl_5]•H_2O$, and experimental PXRD pattern for $[H_2BPZ][SbCl_5]$ (top).

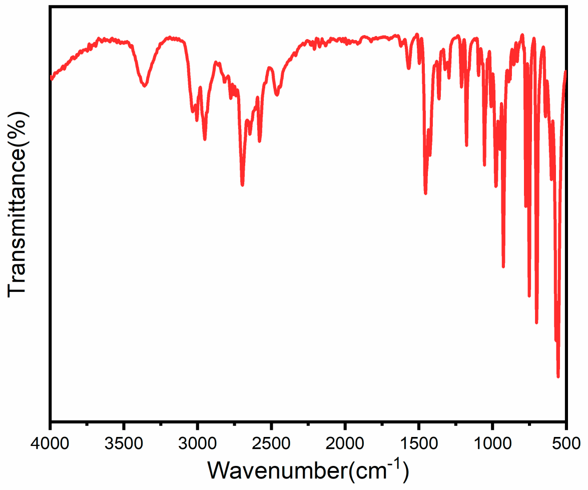

Figure S7. FTIR spectrum of [H_2BPZ][$SbCl_5$]•H_2O crystal powder at RT.
